# Investigating the association between polymorphisms in connective tissue growth factor and susceptibility to colon carcinoma

**DOI:** 10.3892/mmr.2014.3083

**Published:** 2014-12-11

**Authors:** ABRAR AHMAD, SHLEAR ASKARI, RAHEL BEFEKADU, VICTORIA HAHN-STRÖMBERG

**Affiliations:** 1 Department of Clinical Medicine, Örebro University, Örebro 701 81, Sweden; 2 Department of Laboratory Medicine, Section for Transfusion Medicine, Örebro University Hospital, Örebro 701 85, Sweden

**Keywords:** genotyping, clinicopathological parameters, pyrosequencing

## Abstract

There have been numerous studies on the gene expression of connective tissue growth factor (CTGF) in colorectal cancer, however very few have investigated polymorphisms in this gene. The present study aimed to determine whether single nucleotide polymorphisms (SNPs) in the CTGF gene are associated with a higher susceptibility to colon cancer and/or an invasive tumor growth pattern. The CTGF gene was genotyped for seven SNPs (rs6918698, rs1931002, rs9493150, rs12526196, rs12527705, rs9399005 and rs12527379) by pyrosequencing. Formalin-fixed paraffin-embedded tissue samples (n=112) from patients diagnosed with colon carcinoma, and an equal number of blood samples from healthy controls, were selected for genomic DNA extraction. The complexity index was measured using images of tumor samples (n=64) stained for cytokeratin-8. The images were analyzed and correlated with the identified CTGF SNPs and clinicopathological parameters of the patients, including age, gender, tumor penetration, lymph node metastasis, systemic metastasis, differentiation and localization of tumor. It was demonstrated that the frequency of the SNP rs6918698 GG genotype was significantly associated (P=0.05) with an increased risk of colon cancer, as compared with the GC and CC genotypes. The other six SNPs (rs1931002, rs9493150, rs12526196, rs12527705, rs9399005 and rs12527379) exhibited no significant difference in the genotype and allele frequencies between patients diagnosed with colon carcinoma and the normal healthy population. A trend was observed between genotype variation at rs6918698 and the complexity index (P=0.052). The complexity index and genotypes for any of the studied SNPs were not significantly correlated with clinical or pathological parameters of the patients. These results indicate that the rs6918698 GG genotype is associated with an increased risk of developing colon carcinoma, and genetic variations at the rs6918698 are associated with the growth pattern of the tumor. The present results may facilitate the identification of potential biomarkers of the disease in addition to drug targets.

## Introduction

Connective tissue growth factor (CTGF), also termed CCN-2 (cysteine rich 61/connective tissue growth factor/nephroblastoma), is a prototypical member of the CCN family. CTGF, similar to other CCN family members, is recognized for its diverse role in cellular processes, including cell proliferation, development, adhesion, angiogenesis, migration and tumorigenesis (1–4). Previous studies have indicated that CTGF is activated by basic fibroblast growth factor (bFGF) and vascular endothelial growth factor (VEGF) (3,4). One of the principal regulators of CTGF production is transforming growth factor β (TGF-β), which functions in tumor initiation and progression (5,6).

*In vitro* studies have indicated that, when the functional effect of CTGF is blocked by antagonists, the proliferation and migration of endothelial cells is reduced (7). Overproduction of CTGF is implicated in fibroproliferative diseases such as pulmonary fibrosis, systemic sclerosis and liver cirrhosis (8–12). Due to diverse autocrine and paracrine actions, CTGF can have negative effects on normal physiological functions, which implicates CTGF as a potential target for therapeutic purposes (8).

The gene expression of CTGF and its association with cancer development has been studied in various cancers, including colorectal cancer (CRC), and CTGF is considered a prognostic marker in multiple types of human carcinoma (13–17). However, a consensus has not been reached on the role of CTGF in tumorigenesis. In studies by Jacobson and Cunningham (4), Zhen *et al* (18) and Ladwa *et al* (19), CTGF was demonstrated to produce opposing effects in different tumor types, and even within the same type of tumor, which can be categorized into three forms: ‘Oncogenic’, ‘tumor suppression’ and ‘complex’ with both properties. Due to the aberrant expression levels in different types of tumor, the overall role of the CCN protein family members in cancer remains unclear (11,20–22).

Studies investigating polymorphisms in growth factor and other genes demonstrate that they have the ability to induce prominent changes in normal functions via the alteration of transcription sites (23,24). Various genotypes that are changed as a result of polymorphisms are involved in different pathological conditions, and can provide information regarding the susceptibility, severity and prognosis of disease (21). For example, CTGF polymorphisms have been overrepresented in patients with systemic sclerosis, hepatic fibrosis and diabetes mellitus nephropathy; however, there have been few conclusive studies on the function of CTGF SNPs in disease susceptibility (9,11,12). Genetic variations in CTGF are rarely used in clinical decision-making, as there are very few studies concerning CTGF polymorphisms in cancer.

Tumor growth and size are important variables for the prognosis of CRC. Various techniques have been introduced for the analysis of tumor growth in different types of carcinoma, but a single widely-accepted set of criteria for grading is required (25). The majority of grading systems stratify a tumor semi-quantitatively into 3–4 grades, in which 1 indicates a high level of differentiation and 4 indicates poor differentiation (26). The infiltrative pattern of a tumor can be distinguished by its invasive front, which can aid prognosis (27). The invasive front is a term used to describe the level of tumor growth into adjacent tissues. The invasive front can be categorized as expansive and infiltrative, in which the infiltrative growth pattern has an irregular invasive front and poorer prognosis, while the expansive growth pattern has a smooth invasive front (28,29). In 2008, a computer software-based technique for measuring the invasiveness of tumors in CRC was introduced by Franzén *et al* (30) in which they quantitatively scored tumors on a scale of 1–5, and labelled the measurement as the complexity index (CI). A grade 1 tumor was defined as having a smooth invasive front, while a grade 5 tumor was defined as having a highly irregular tumor front in addition to separate tumor cells and cell clusters. This classification was based on the fractile dimensions and the number of tumor cells (30).

Tumor growth depends upon numerous proteins that are important in maintaining the morphology of tissues and affect invasion and metastasis (10,31). Tumors present limitations with respect to therapy, due to their infiltrative nature, which inhibits complete resection and contributes to tumor recurrence and resistance to radio- and chemotherapy (32). Previous studies have demonstrated that the complexity index of a tumor is associated with tumor wall penetration, progression and stage (33,34). As the action of CTGF in the metastasis, proliferation and migration of tumor cells is well-established (2,35,36), it was assumed in the current study that genetic variation is able to cause changes in the tumor phenotype, which can affect the CI of the tumor.

Polymorphic alleles of various growth factors such as VEGF, TGF-β and bFGF have been well-defined with respect to their potential role in CRC development (37–39). Currently, a limited number of studies investigating the role of CTGF in CRC have been published, and genetic variations in this gene have yet to be studied in patients with CRC. The aim of the current study was to assess the following SNPs in the CTGF gene in patients diagnosed with CRC: rs6918698, rs1931002, rs9493150, rs12526196, rs12527705, rs9399005 and rs12527379. This was then compared with the normal healthy population, in addition to comparing the SNPs in patients with different clinicopathological parameters, including age, gender, tumor wall penetration, lymph node metastasis, systemic metastasis, localization and tumor differentiation. Five-year survival data from the patients associated with genetic variations was produced, in order to gain information regarding the role of CTGF and genotypes associated with the risk of development of CRC.

## Materials and methods

### Patient material

A total of 112 formalin-fixed paraffin-embedded (FFPE) samples from patients diagnosed with CRC at the Department of Laboratory Medicine, section for Pathology, Örebro University Hospital (Örebro, Sweden) between 2004 and 2009 were selected. Rectal carcinoma samples were not used, as rectal carcinoma is often treated with radiation prior to surgery, which can alter the morphological and genetic characteristics of the tumor. Blood samples from 112 blood and plasma donors were used as controls. An initial screening of patient and control samples (n=67 of each) was performed for seven known SNPs in CTGF (rs6918698, rs1931002, rs9493150, rs12526196, rs9399005, rs12527379 and rs12527705). Following evaluation of the results, samples that showed significance or a trend toward significant association between polymorphism and disease were processed, resulting in 112 CRC samples and 112 normal blood samples. These samples (n=112) were analyzed for the following SNPs: rs6918698, rs1931002, rs9493150, rs12526196 and rs12527705. Two SNPs (rs9399005 and rs12527379) were analyzed in 67 patient and 67 control samples. The samples were collected from both males and females. The present study was approved by the Ethical Review Board, EPN (Uppsala, Sweden).

### DNA extraction

The tumor area was outlined by an experienced morphologist (Hahn-Strömberg). Depending upon the size of the tumor samples, 1–2 tissue punches of 2-mm diameter were obtained from the tumor area in the FFPE blocks. Genomic DNA was extracted from this area using a NucleoSpin^®^ FFPE DNA kit (Macherey-Nagel GmbH, Düren, Germany) according to the manufacturer’s instructions. Genomic DNA from blood and plasma donors was extracted using a NucleoSpin^®^ Blood DNA Extraction kit (Macherey-Nagel GmbH) and the concentration and quality of the DNA was analyzed using a NanoDrop 1000 spectrophotometer (Thermo Fisher Scientific, Wilmington, DE, USA).

### Primer designin and optimization

Primers were designed using PyroMark Assay DesignSoftware, version 2.0 (Qiagen, Hilden, Germany). The primers were optimized by polymerase chain reaction (PCR) at different temperatures and MgCl_2_ concentrations. The primer sequences (forward, reverse and sequencing primers) and their annealing temperatures are presented in Table I.

### PCR

A master mix was prepared, containing the following reagents: Reverse and forward primers (0.25 μM) (Biomers.net GmbH, Ulm, Germany) KAPA2G Buffer M (1X), KAPA MgCl_2_ (1 mM), KAPA dNTP Mix (200 μM), KAPA2G Fast HotStart DNA Polymerase (1 U) (KAPA2G Fast HotStart PCR kit, KK5512; Kapa Biosystems, Inc., Wilmington, MA, USA) and genomic DNA (90–100 μg). PCR reactions were conducted in an ABI 2720 Thermal Cycler (Life Technologies, Carlsbad, CA, USA) in three steps as follows: (i) Denaturation at 95°C for 10 min; (ii) 49 cycles with denaturation at 94°C for 45 sec, annealing temperature (according to optimized annealing temperature of primers) for 30 sec, and extension at 72°C for 30 sec; (iii) an extension was completed at 72°C for 7 min.

### Gel electrophoresis

High-resolution agarose (Sigma-Aldrich, St. Louis, MO, USA) was added to 1X TBE (Tris base, acetic acid and EDTA) buffer solution to produce a 2% solution of agarose. A MassRuler Low Range DNA Ladder (Thermo Fisher Scientific, Pittsburgh, PA, USA) was used to compare the amplicon sizes following agarose gel separation. The PCR products were visualized using a UV Transilluminator (Bio-Rad Laboratories AB, Sundbyberg, Sweden).

### Polymorphism screening by pyrosequencing

Pyrosequencing was performed using a PyroMark Q96 ID sequencing and quantification platform (Qiagen AB, Sollentuna, Sweden). A master mix solution of Streptavidin Sepharose High Performance Beads (GE Healthcare, Uppsala, Sweden) was prepared by diluting sepharose beads in ultra-pure Milli-Q water and 1X binding buffer (1 mM/l EDTA, 0.1% Tween 20, 2 M/l NaCl, 10 mM/l Tris-HCl, Milli-Q water; pH 7.6). The streptavidin solution was added to a 96-well PCR plate, followed by the addition of the amplified PCR product from each sample. Another solution was prepared for the sequencing primer by diluting it to 0.5 μM with 1X annealing buffer (2 mM/l magnesium acetate, 20 mM/l Tris-acetate; pH 7.6) at a ratio of 1:249 and adding it to a PSQ96 well plate. A PyroMark Q96 Vacuum Workstation (Qiagen, Hilden, Germany) was used to purify the biotinylated PCR product. Following purification, the PSQ96 plate was heated at 80°C for 2 min and was left to cool at room temperature for 10 min. The polymorphisms were analyzed using PyroMark ID software, version 1.0 (Qiagen AB, Upsala, Sweden). The substrate mixture, enzymes and dNTPs were added to the cartridge according to calculation generated by the PyroMark Q 96 ID system (Qiagen AB, Uppsala, Sweden). A PyroMark Gold Q96 Reagent kit (Qiagen AB, Uppsala, Sweden) was used according to manufacturer’s instructions.

### CI

To calculate the CI, 64 tumor samples were randomly selected for computer image analysis from one patient group used for the CTGF SNP study. Slide preparations, including sectioning, staining and image processing were performed using the methodology as described by Franzén *et al* (30). In brief, images from the invasive front of the tumor area were captured using a Leica DC200 digital camera mounted on a Leica DMRXE microscope with 10X objective lens (Leica Microsystems GmbH, Wetzlar, Germany). From each sample, an average of 7 (range of 5–10) images were captured. The number of images depended upon the length of the tumor-stromal area. Images were adjusted so that the tumor area appeared black and the background white. These images were used to calculate the number of free tumor cells and tumor cell clusters. The black color was then removed so that only the outline of tumor remained (40). Using the tumor outline image, the fractile dimensions were calculated using various software programs; Adobe Photoshop, version 7.0 (Adobe Systems, Inc., San Jose, CA, USA) with the Fovea Pro (Reindeer Graphics, Inc., Asheville, NC, USA) was used for the black/white and the tumor outline images, and ImageJ software (http://imagej.nih.gov/ij/) was used to calculate the fractal dimension value. The CI (ranges 1–5) was obtained by calculating the mean value of these parameters.

### Statistical analysis

SPSS, version 20 (IBM SPSS, Armonk, NY, USA) was used for statistical analysis. Continuous variables were measured as the mean and standard deviations. Univariant binary logistic regression was applied to determine different SNPs as risk factors for CRC. The Pearson’s χ^2^ test was used where required to assess the data trends. The CI association was measured using the Fisher’s exact test. Survival was analyzed using the Kaplan-Meier’s test. P≤0.05 was considered to indicate a statistically significant difference.

## Results

### Genetic analysis

**Figure 1:**
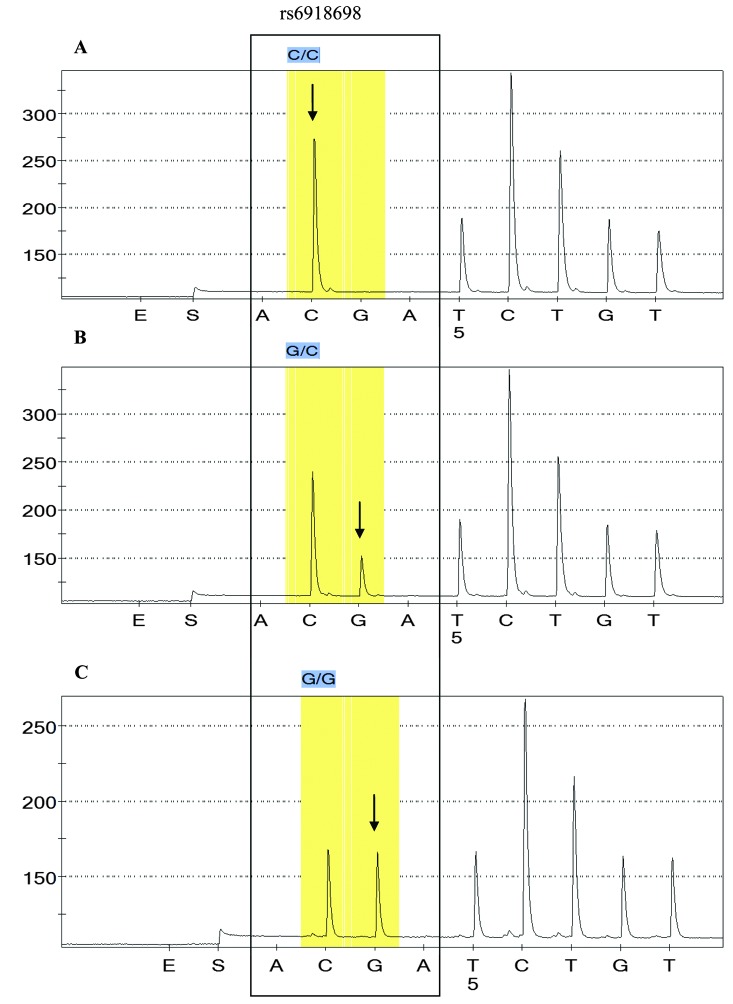
Illustration of rs6918698 single nucleotide polymorphism pyrograms in connective tissue growth factor. (A) Wild type CC genotype. The polymorphism is at the same codon (arrow), in (B) one C is replaced with G and in (C) CC is replaced by GG.

The allele frequencies and genotype distributions in the patient and control samples are summarized in Table II. The association between CTGF polymorphisms and occurrence of CRC was compared with the clinicopathological parameters described below. A significant difference in the number of samples with the rs6918698 GG genotype was established between the CRC and the control group samples (P=0.05; Table II). All three genotypes in colon carcinoma sample (CC, GC and GG) were correlated with respective genotypes in normal samples. GG genotype was significantly different in tumor samples as compared with normal samples (P=0.05). No significant difference was identified in genotypic frequencies of GC between normal and CRC samples (P=0.833). CC being a wild type, was considered as a referent. Fig. 1 indicates the different genotypes in rs6918698.

For the rs1931002, rs9493150, rs12526196, rs12527705, rs9399005 and rs12527379 SNPs, no significant association was identified between patients and normal controls (Table II). Clinicopathological parameters, including age, gender, localization and tumor differentiation were analyzed but did not present any significant differences. Tumor penetration (T), lymph node involvement (N) and distance metastasis (M) were also analyzed, but no significant differences were identified (P=0.567, P=0.951 and P=1.00 respectively).

**Figure 2:**
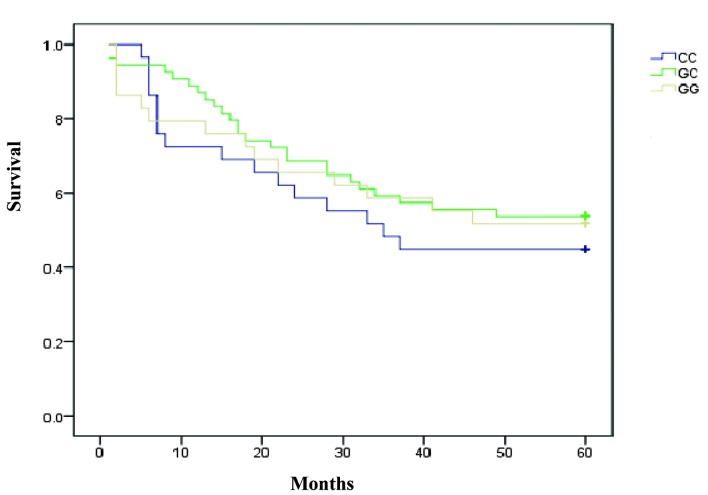
Survival curve presenting the different genotypes associated with rs6918698. There was no significant association between any genotype with survival.

The 5-year survival data of the patients indicated no significant association between the survival time and the CTGF polymorphisms studied. Statistical results of the survival test were as follows: rs6918698, P=0.668; rs1931002, P=0.367; rs9493150, P=0.409; rs12526196, P=0.868; rs12527705, P=0.489; rs9399005, P=0.123; and rs12527379, P=0.599 (Table III, Fig. 2).

### Patient clinicopathological data

SNPs in the CTGF gene were determined by pyrosequencing. A total of 224 samples were used in the current study, consisting 112 samples from patients diagnosed with CRC between 2004 and 2009, and 112 samples from healthy blood and plasma donors. Of the patients with CRC, 67 (60%) were male and 45 (40%) were female. There were 7 (6.2%) patients <60 years of age and 105 (93.7%) that were >60. Regarding tumor wall penetration (T), 4 (3.5%) were classified as T1; 18 (16%) T2; 76 (68%) T3; and 14 (12.5%) T4. For lymph node metastasis (N), 62 (55.3%) patients presented N0 tumors; 32 (28.5%), N1; and 17 (15.1%), N2. With regards to metastasis (M), 8 (7.1%) patients were classified as M1, while the remaining 104 (92.8%) were at the Mx stage. For tumor differentiation, 19 (16.9%) were low; 71 (63.4%) were moderate; and 18 (16%) were at the high differentiation stage.

The tumors were divided into two localizations; right and left colon. There were 73 (65.1%) right-colon, and 39 (34.8%) left-colon tumors. The survival data demonstrated that 57 (50.8%) patients survived >5 years and 55 (49.1%) died within 5 years of CRC diagnosis (Tables III and IV).

### SNP and HapMap comparison

When comparing the SNP frequencies to the HapMap data (http://hapmap.ncbi.nlm.nih.gov/cgi-perl/snp_details_phase3?name=rs6918698&source=hapmap28_B36&tmpl=snp_details_phase3) for the Central European population, a noticeable difference was observed in genotype frequencies between the tumor, normal and HapMap data in all SNPs (rs6918698, rs1931002, rs9493150, rs12526196, rs12527705, rs9399005 and rs12527379 (Table V).

### CI

**Figure 3:**
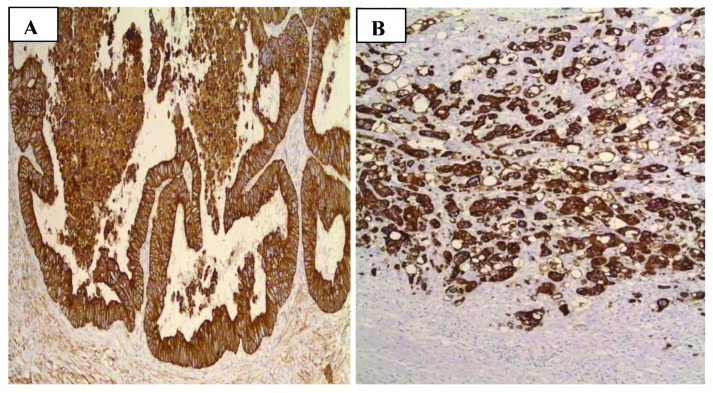
Human colon biopsies exhibiting tumor growth patterns in colon carcinoma. (A) Expansive tumor growth with smooth invasive front (CI=1). (B) The infiltrative growth pattern with highly coarse invasive front and dispersed tumor cells (CI=5). CI, complexity index.

To assess the CI, images of 64 tumor samples were analyzed (Fig. 3) and the clinicopathological parameters and genetic variation were compared in the seven SNPs, rs6918698, rs1931002, rs9493150, rs12526196, rs9399005, rs12527379 and rs12527705. The CI data was divided into 3 groups: Low (CI=1), medium (CI=2,3) and high (CI=4,5). A trend was observed between the genetic variation at SNP rs6918698 and the CI of the tumor (P=0.052). No significant association was identified between the other six SNPs CI of tumor (Table VI). Associations of CI with clinicopathological parameters and CTGF SNPs were as follows: Gender, P=0.885; age, P=0.321; T, P=0.737; N, P=0.949; M, P=0.1; localization, P=0.345; differentiation, P=0.280; rs6918698, P=0.052; rs1931002, P=0.453; rs9493150, P=0.370; rs12526196, P=0.285; rs12527705, P=889; rs9399005, P=0.959; and rs12527379, P=0.506.

## Discussion

CTGF is a multicellular protein involved in promoting endothelial cell growth, adhesion and angiogenesis. CTGF has been studied for its role in various diseases such as sclerosis, kidney fibrosis, hepatic fibrosis, and numerous cancers, including CRC (9,12,17,19,22). Previously, only gene expression of CTGF has been analyzed in CRC, thus very little is known about the role of CTGF polymorphisms in this disease (19). In the current study, seven SNPs (rs6918698, rs1931002, rs9493150, rs12526196, rs12527705, rs9399005 and rs12527379) were investigated in the CTGF gene and correlated to the different clinicopathological parameters. Notably, it was demonstrated that the GG genotype of rs6918698 was significantly associated with an increased susceptibility to developing CRC (P=0.05). The other two genotypes, GC and CC in SNP rs6918698, indicated no statistical significance. It may be hypothesized that the C allele is a protective, and substitution with the G allele leads to an increased risk for disease development. Similar results were indicated by Fonseca *et al* (41) who demonstrated that CTGF gene expression is greater when the C is substituted for a G allele in systemic sclerosis (41). This effect may be due to the association between certain genotypes being more frequently involved in transcription and stabilization of mRNA than others in different genes. Previous studies have shown the differential expression of polymorphic variants of the same genes (e.g. myeloperoxidase G463A and TGFβ C1815T) (42–44). Similar findings were made by Ladwa *et al* (19) indicating that gene polymorphisms can change their gene expression behavior.

The polymorphisms rs1931002, rs9493150, rs12526196, rs12527705, rs9399005 and rs12527379 were not observed to be correlated with cancer risk, as most of the SNPs produced silent mutations. Polymorphisms in coding regions likley alter the protein function, whereas polymorphisms in the gene regulatory regions may have an effect on gene expression. Pivovarova *et al* (22) studied SNP rs9493150 in pancreatic fibrosis but did not observe any correlation with disease development. Similar results were obtained in a study by Kovalenko *et al* (45) on liver fibrosis, in which the rs9493150 and rs9399005 polymorphisms were not associated with the disease. These studies support the current findings indicating that these are silent polymorphisms. In a French population study, SNP rs9399005 was demonstrated to be significantly associated with systemic sclerosis (9). However, in the current study, this SNP was not observed to be significantly associated with the development of CRC, suggesting that this SNP performs a specific role in sclerosis, but not in CRC. The difference in this finding may be due to the different sample populations and methods used for analysis. Similar results were produced in a study by Dessein *et al* (12), in which CTGF SNPs (rs12526196 and rs1931002) were indicated to serve a significant function in hepatic fibrosis. However, SNP rs12527705 did not present any significant association with tumor growth in CRC in the current study. The resulting proteins of these polymorphisms may have a significant function in fibrosis in organs such as the liver, but are not associated with angiogenesis and tumor growth in CRC.

CTGF has been reported to be involved in binding with TGFβ, thereby enhancing its signalling (45). This demonstrates that polymorphisms are more strongly associated with certain diseases compared with others. In the present study, a high frequency of the rs6918698 GG genotype was identified in patients diagnosed with CRC, but the same SNP studied by Granel *et al* (9) and Robinson *et al* (6) was indicated to not be associated with fibrosis, which supports the idea that polymorphisms have different functions in different diseases.

The frequencies of all the SNP genotypes, in the tumor and normal samples, were compared with the HapMap data of the Central European population (CEU) in the current study. All the studied SNPs presented different frequencies to the CEU data, which may be due to the different population samples; the current study used samples from a Swedish population.

As descibed in earlier studies, little is still known about the role of CCN proteins in cancer, and the results are controversial, thus the role of CTGF in cancer remains undefined. CTGF has an important role in the angiogenesis of breast cancer, and is overexpressed in esophageal adenocarcinoma and CRC (13,17,19,46). Paradoxically, studies by Lin *et al* (47) and Chang *et al* (48) indicated that CTGF inhibits metastasis and that overexpression is associated with high survival and good prognosis in lung adenocarcinoma (47,48). In esophageal carcinoma, this overproduction increases the β-catenin/T-cell factor signalling while opposite results are observed in CRC (47,49). As this divergence is not yet understood, further studies are required.

In the present study, the CI was assessed in 64 tumor samples. The results indicated a trend toward a significant association between CTGF rs6918698 genotype variation and tumor growth pattern (P=0.052). This demonstrates that genetic variation at rs6918698 has an affect on the phenotype of tumors. Previous studies have indicated that when a tumor metastasizes, its phenotype changes; more aggressive tumors have a more irregular invasive front with high CI (33,34); however, conflicting outcomes have been observed by other researchers (40,50). In the present study, polymorphism rs6918698 was associated with a high risk of developing CRC, which indicates its importance in this disease. To confirm any association between rs6918698 genotypes and the growth patterns of tumors, further studies are required in which a larger number of samples must be examined. In the current study, there was no significant association or trend between CI and the remaining six CTGF polymorphisms. All the SNPs were evaluated for any possible association with clinicopathological parameters, including age, gender, tumor wall penetration, lymph node and systemic metastasis, localization and tumor differentiation. No statistically significant correlations were identified with any of these parameters.

Previous studies have demonstrated that integrin-TGFβ is involved in cancer development and fibrosis, and that CTGF is a downstream effector of TGFβ (13,51). It has been indicated that fibrosis can lead to cancer development in various tissues (52), and so polymorphisms in these genes that have an important role in fibrosis should be studied further to clarify their role in cancer development.

In conclusion, the present study was, to the best of our knowledge, the first study conducted in which the association between CTGF polymorphisms, CI and CRC was analyzed. The results, however, did not indicate any significant association between CI, CTGF polymorphism and tumor progression, but a trend was detected between genetic variation at rs6918698 and tumor growth pattern. Another notable finding was that the occurrence of the SNP rs6918698 GG genotype indicated a higher risk of developing CRC. This polymorphism and its association with growth pattern should be investigated in future experiments, using different populations, a larger sample size and different types of tumor, for further understanding of the importance of CTGF SNPs in cancer. This SNP may be a valuable marker in determining risk and progression of different malignant diseases, and a critical step in the future treatment of CRC that could be targeted for chemotherapy.

## Figures and Tables

**Table I:** Forward, reverse and sequencing primers used to analyze seven single nucleotide polymorphisms in connective tissue growth factor.

SNP number	Primers	Annealing temperature (°C)	Amplicon length (bp)
rs6918698	F: GGGGCAGATTTCCAAAACTCTT^a^ R: TGGATCCCTTTTTCTGGAAACA S: AACATTGATGGCCACT	54	112
rs1931002	F: CCCATAGGCATGGTTATTTAAAGA R: AGCAAACTTGGTAGCCAGTATGT^a^ S: TTTAGAAACTCTTTGGATGA	54	116
rs9493150	F: TCAGAGCATGGGTTCAAGATAA^a^ R: CAGGCTGTTTTCAAATGATAAATC S: CCGATCTTTGCACCA	53	111
rs12526196	F: AGAGGAAAATCGTTCACCATTTTA R: TACATGCAACACACATCGAATCTC^a^ S: AAGACAACACTGAATATACA	52	113
rs12527705	F: CAATGGTGCTCCTCATTTCTT^a^ R: GGATTCAAAGCAATAGACATGTAG S: GCAATAGACATGTAGACCC	52	94
rs9399005	F: TGATGTGAAGGGTTGGAAACTAA^a^ R: TCAGTCTCCATTAACCCTGTTGTA S: GCATTTGTACCTCTCTGG	54	93
rs12527379	F: AGCTTTCTCCCTCTCTCCTTTAA R: CCTCTCTCTCTGCCATGTGTAGTT^a^ S: ATGTTGTTAATGGAATGC	54	111

a Biotinylated primer; F, forward; R, reverse; S, sequencing primer; SNP, single nucleotide polymorphis; bp, base pairs.

**Table II:** Genotypes in connective tissue growth factor and their association as risk factors for colorectal cancer.

CTGF SNPs	Genotype	Total CRC and control samples (% all samples)	Control (% controls)	CRC (% CRC)	P-value	OR	95% CI
rs6918698	CC	64 (28.6)	35 (31.2)	29 (25.9)	0	1.00	(Referent)
	GC	115 (51.3)	61 (54.5)	54 (48.2)	0.833	1.068	0.579–1.973
	GG	45 (20.1)	16 (14.3)	29 (25.9)	0.050	2.187	0.999–4.79
rs1931002	GG	198 (88.4)	98 (87.5)	100 (89.3)	0	1.00	(Referent)
	GA	23 (10.3)	13 (11.6)	10 (8.9)	0.524	0.75	0.316–1.8
	AA	3 (1.3)	1 (0.9)	2 (1.8)	0.585	1.96	0.175–21.966
rs9493150	CC	129 (57.6)	67 (59.8)	62 (55.4)	0	1.00	(Referent)
	GC	81 (36.2)	40 (35.7)	41 (36.6)	0.718	1.108	0.635–1.93
	GG	14 (6.2)	5 (4.5)	9 (8)	0.255	1.945	0.618–6.122
rs12526196	TT	183 (81.7)	94 (83.9)	89 (79.5)	0	1.00	(Referent)
	TC	34 (15.2)	18 (16.1)	16 (14.3)	0.866	0.939	0.451–1.954
	CC	7 (3.1)	0 (0)	7 (6.2)	0.999	N/A	N/A
rs12527705	TT	162 (72.3)	78 (69.6)	84 (75.0)	0	1.00	(Referent)
	AT	57 (25.4)	34 (30.3)	23 (20.5)	0.137	0.628	0.341–1.159
	AA	5 (2.2)	0 (0)	5 (4.4)	0.9999	N/A	N/A
rs9399005	GG	63 (47)	34 (50.7)	29 (43.3)	0	1.00	(Referent)
	GA	61 (45.5)	29 (43.3)	32 (47.8)	0.474	1.294	0.639–2.62
	AA	10 (7.5)	4 (6)	6 (9)	0.415	1.759	0.452–6.843
rs12527379	GG	41 (30.6)	17 (25.4)	24 (35.8)	0	1.00	(Referent)
	GA	65 (48.5)	37 (55.2)	28 (41.8)	0.123	0.536	0.243–1.183
	AA	28 (20.9)	13 (19.4)	15 (22.4)	0.683	0.817	0.31–2.152

CTGF, connective tissue growth factor; CRC, colorectal cancer; OR, odds ratio; CI, confidence interval; N/A, not applicable; SNP, single nucleotide polymorphism.

**Table III:** Association between different single nucleotide polymorphisms in connective tissue growth factor and patient survival.

CTGF SNPs	Genotype	Survival P-value Kaplan-Meier’s test	P-value Cox-regression test	OR	95% CI
rs6918698	CC	0.668		1.00	Referent
	GC		0.374	0.752	1.402–1.410
	GG		0.612	0.83	0.405–1.702
rs1931002	GG	0.367		1.00	Referent
	GA		0.174	0.445	0.139–1.428
	AA		0.911	1.119	0.155–8.104
rs9493150	CC	0.409		1.00	Referent
	GC		0.187	1.455	0.834–2.538
	GG		0.709	1.199	0.462–3.115
rs12526196	TT	0.868		1.00	Referent
	TC		0.62	0.817	0.368–1.815
	CC		0.901	1.067	0.383–2.971
rs12527705	TT	0.489		1.00	Referent
	AT		0.876	0.938	0.421–2.089
	AA		0.266	1.98	0.594–6.608
rs9399005	GG	0.123		1.00	Referent
	GA		0.48	0.772	0.377–1.583
	AA		0.132	2.182	0.791–6.021
rs12527379	GG	0.599			
	GA		0.321	0.682	0.320–1.452
	AA		0.592	0.768	0.330–1.881

SNP, single nucleotide polymorphism; CTGF, connective tissue growth factor; OR, odds ratio; CI, confidence interval.

**Table IV:** Clinicopathological data of the patients diagnosed with colorectal cancer.

Parameter studied	N (% of total)
Age	
≤60 years	7 (6.2)
>60 years	105 (93.7)
Gender	
Male	67 (60)
Female	45 (40)
Tumor penetration	
T1	4 (3.5)
T2	18 (16)
T3	76 (68)
T4	14 (12.5)
Lymph node metastasis	
N0	62 (55.3)
N1	32 (28.5)
N2	17 (15.1)
Metastasis	
M1	8 (7.1)
Mx	104 (92.8)
Differentiation	
Low	19 (16.9)
Medium	71 (63.4)
High	18 (16)
Localization	
Right colon	73 (65.1)
Left colon	39 (34.8)
Survival	
Survived	57 (50.8)
Died	55 (49.1)

CRC, colorectal cancer; N, number of samples.

**Table V:** Frequencies of polymorphisms in connective tissue growth factor from the sampled patients compared with HapMap data for the central European population.

	Tumor		Normal		HapMap	
			
Genotype	Frequency	Number	Frequency	Number	Frequency	Number
SNP rs6918698						
CC	0.26	29	0.31	35	0.21	13
GC	0.48	54	0.54	61	0.44	27
GG	0.26	29	0.14	16	0.35	22
rs1931002						
GG	0.89	100	0.87	98	0.72	47
GA	0.09	10	0.12	13	0.23	15
AA	0.018	2	0.01	1	0.05	3
rs9493150						
CC	0.55	62	0.6	67	0.5	56
GC	0.37	41	0.36	40	0.4	45
GG	0.08	9	0.04	5	0.1	12
rs12526196						
TT	0.80	89	0.84	94	0.89	100
TC	0.14	16	0.16	18	0.09	10
CC	0.06	7	0	0	0.02	2
rs12527705						
TT	0.75	84	0.70	78	N/A	N/A
AT	0.21	23	0.30	34	N/A	N/A
AA	0.04	5	0	0	N/A	N/A
rs9399005						
GG	0.43	29	0.51	34	0.56	63
GA	0.48	32	0.43	29	0.34	38
AA	0.09	6	0.06	4	0.106	12
rs12527379						
GG	0.36	24	0.25	17	0.33	37
GA	0.42	28	0.55	37	0.56	63
AA	0.22	15	0.19	13	0.11	13

N/A, not applicable; HapMap, International HapMap Project data.

**Table VI:** Association of complexity index with clinicopathological parameters of colorectal cancer and single nucleotide polymorphisms in connective tissue growth factor.

Parameters	Low CI (% of total)	Medium CI (% of total)	High CI (% of total)	P-value
Gender				
Male	8 (12.5)	22 (34.49)	10 (15.6)	0.885
Female	6 (9.4)	13 (20.3)	5 (7.8)	
Age				
<60 years	0 (0)	4 (6.2)	0 (0)	0.321
>60 years	14 (21.9)	31 (84.4)	15 (23.4)	
Tumor stage (T)				
T1	1 (1.6)	2 (3.1)	0 (0)	0.737
T2	4 (6.2)	4 (6.2)	2 (3.1)	
T3	8 (12.5)	25 (39.1)	12 (18.8)	
T4	1 (1.6)	4 (6.2)	1 (1.6)	
Lymph node metastasis (N)				
N0	8 (12.5)	20 (31.7)	8 (12.5)	0.949
N1	3 (4.8)	9 (14.3)	3 (4.8)	
N2	2 (3.1)	6 (9.5)	4 (6.2)	
Metastasis (M)				
Mx	13 (20.3)	32 (50.0)	14 (21.9)	1
M1	1 (1.6)	3 (4.7)	1 (1.6)	
Localization				
Right colon	9 (14.1)	24 (37.5)	13 (20.3)	0.345
Left colon	5 (7.8)	11 (17.2)	2 (3.1)	
Differentiation				
Low	3 (4.8)	3 (4.8)	4 (6.3)	0.280
Medium	7 (11.1)	26 (41.3)	8 (12.5)	
High	4 (6.3)	6 (9.5)	2 (3.1)	
rs6918698				
CC	3 (4.7)	11 (17.2)	3 (4.7)	0.052
GC	3 (4.7)	17 (26.6)	10 (15.6)	
GG	8 (12.5)	7 (10.9)	2 (3.1)	
rs1931002				
GG	14 (21.9)	35 (54.7)	14 (21.9)	0.453
GA	0 (0)	0 (0)	1 (1.6)	
rs9493150				
CC	11 (17.2)	19 (29.7)	9 (14.1)	0.370
GC	2 (3.1)	13 (20.3)	6 (9.4)	
GG	1 (1.6)	3 (4.7)	0 (0)	
rs12526196				
TT	13 (20.3)	27 (42.2)	13 (20.3)	0.285
TC	1 (1.6)	6 (9.4)	0 (0)	
CC	0 (0)	2 (3.1)	2 (3.1)	
rs12527705				
TT	9 (14.1)	25 (39.1)	11 (17.2)	0.889
AT	4 (6.2)	9 (14.1)	3 (4.7)	
AA	1 (1.6)	1 (1.6)	1 (1.6)	
rs9399005				
GG	5 (7.8)	15 (23.4)	6 (9.4)	0.959
GA	7 (10.9)	17 (26.6)	8 (12.5)	
AA	2 (3.1)	3 (4.7)	1 (1.6)	
rs12527379				
GG	8 (12.5)	11 (17.2)	4 (6.2)	0.506
GA	4 (6.2)	16 (25.0)	7 (10.9)	
AA	2 (3.1)	8 (12.5)	4 (6.2)	

CI, complexity index.
